# DNA-methylation in *C1R* is a prognostic biomarker for acute myeloid leukemia

**DOI:** 10.1186/s13148-015-0153-6

**Published:** 2015-11-04

**Authors:** Tanja Božić, Qiong Lin, Joana Frobel, Stefan Wilop, Melanie Hoffmann, Carsten Müller-Tidow, Tim H. Brümmendorf, Edgar Jost, Wolfgang Wagner

**Affiliations:** Helmholtz-Institute for Biomedical Engineering, Stem Cell Biology and Cellular Engineering, University Hospital of the RWTH Aachen, Pauwelsstrasse 20, 52074 Aachen, Germany; Institute for Biomedical Engineering - Cell Biology, University Hospital of the RWTH Aachen, Aachen, Germany; Department of Hematology, Oncology, Hemostaseology and Stem Cell Transplantation, University Hospital of the RWTH Aachen, Aachen, Germany; Department of Hematology and Oncology, University of Halle, Halle, Germany

**Keywords:** DNA-methylation, Epigenetic, Leukemia, AML, *C1R*, Survival, Biomarker, Prognosis, TCGA

## Abstract

**Background:**

Epigenetic aberrations play a central role in the pathophysiology of acute myeloid leukemia (AML). It has been shown that molecular signatures based on DNA-methylation (DNAm) patterns can be used for classification of the disease. In this study, we followed the hypothesis that DNAm at a single CpG site might support risk stratification in AML.

**Findings:**

Using DNAm profiles of 194 patients from The Cancer Genome Atlas (TCGA), we identified a CpG site in complement component 1 subcomponent R (*C1R*) as best suited biomarker: patients with higher methylation at this CpG site (>27 % DNAm) reveal significantly longer overall survival (53 versus 11 months; *P* < 0.0001). This finding was validated in an independent set of 62 DNAm profiles of cytogenetically normal AML patients (*P* = 0.009) and with a region-specific pyrosequencing assay in 84 AML samples (*P* = 0.012). DNAm of *C1R* correlated with genomic DNAm and gene expression patterns, whereas there was only moderate association with gene expression levels of *C1R*. These results indicate that DNAm of *C1R* is a biomarker reflecting chromatin reorganization rather than being of pathophysiological relevance per se. Notably, DNAm of *C1R* was associated with occurrence of specific genomic mutations that are traditionally used for risk stratification in AML. Furthermore, DNAm of *C1R* correlates also with overall survival in several other types of cancer, but the prognostic relevance was less pronounced than in AML.

**Conclusions:**

Analysis of DNAm at *C1R* provides a simple, robust, and cost-effective biomarker to further complement risk assessment in AML.

**Electronic supplementary material:**

The online version of this article (doi:10.1186/s13148-015-0153-6) contains supplementary material, which is available to authorized users.

## Findings

Risk assessment is relevant for the choice of therapeutic regimen in acute myeloid leukemia (AML). It is usually based on many parameters including age, white blood cell count, cytogenetic abnormalities, and specific mutations [1]. Epigenetic modifications, such as DNA-methylation (DNAm) changes, seem to play a critical role in pathogenesis of AML [2]. Various studies demonstrated that DNAm patterns can discriminate subgroups of patients with different clinical outcomes [3–7]. So far, these approaches utilized a combination of many differentially methylated regions and therefore require DNAm profiles based on microarray or sequencing technology. In contrast, a region-specific assay would be much faster, economic, and easier to interpret. We have recently described that aberrant hypermethylation at a specific region of the *de novo* methyltransferase *DNMT3A* is associated with poor prognosis in AML, but this association was only significant for patients without genomic mutations in *DNMT3A* because both modifications seem to have a similar molecular and clinical sequel [8]. This exemplifies that identification of simple epigenetic markers is hampered by the high molecular and clinical heterogeneity of the disease. Despite these hurdles, we followed the hypothesis that DNAm at a unique CpG site might provide a robust biomarker to further support risk assessment of AML.

### Selection of CpG sites that are indicative for overall survival in AML

**Fig. 1:**
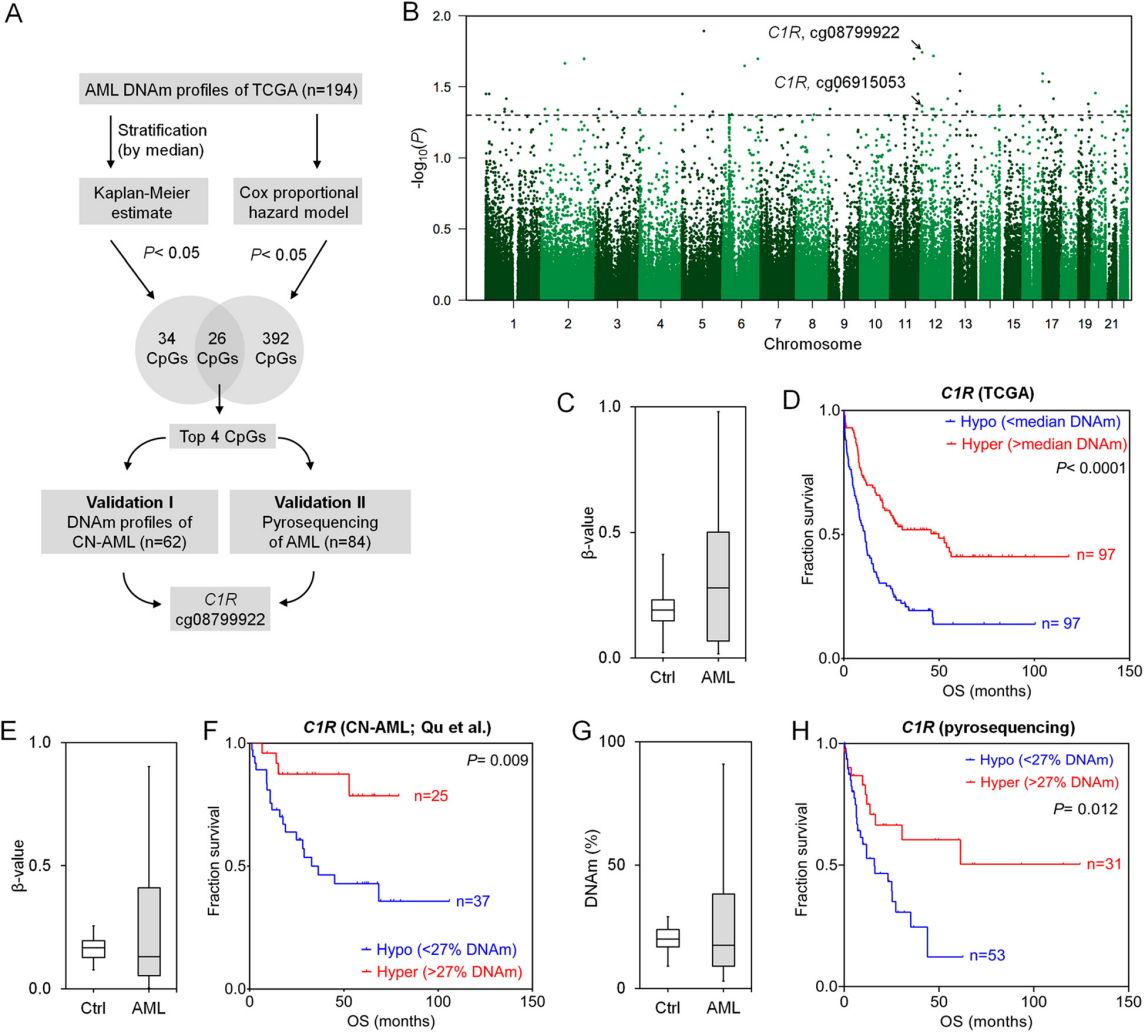
DNA-methylation at *C1R* has prognostic relevance in AML. **a** Scheme for selection of the relevant CpG site. **b** For Kaplan-Meier (K-M) estimation of overall survival (OS) in 194 AML patients, [9] the DNAm levels for each of the 390,000 CpGs were stratified by their median DNAm level. The *Manhattan plot* demonstrates that the 60 CpGs with adjusted *P* < 0.05 (*dashed line*) are distributed over different chromosomes. **c** Beta-value distribution at the CpG site cg08799922 (*C1R*) in 656 healthy controls (GSE40279) [19] and 194 AML samples (TCGA) [9]. **d** K-M plot of TCGA samples classified by median DNAm level at cg08799922 (*C1R*; median DNAm level 27 %; *P* < 0.0001). **e** Beta-value distribution of DNAm in *C1R* of 10 healthy controls and 62 cytogenetic normal AML (CN-AML) samples (GSE58477) [13]. **f** Validation of prognostic relevance of cg08799922 for OS in this dataset (*P* < 0.009; classified by DNAm level of 27 %). **g** Beta-value distribution of DNAm in *C1R* of 40 healthy controls and 84 AML samples analyzed by pyrosequencing. **h** K-M plot of these AML samples upon classification by 27 % DNAm level at the relevant CpG site in *C1R* (*P* < 0.012)

For initial selection of prognostic relevant CpGs, we utilized 194 DNAm profiles of AML patients from The Cancer Genome Atlas (TCGA) [9]. For each CpG site, the samples were stratified by its median DNAm level to estimate association with overall survival (OS) using the Kaplan-Meier (K-M) method (Fig. 1a). Only 60 CpGs revealed significant association upon adjustment for multiple testing (adjusted *P* < 0.05; Fig. 1b). Alternatively, we selected 418 CpGs with significant association with OS by COX-regression analysis (adjusted *P* < 0.05; Additional file 1: Figure S1). Only 26 CpGs were in the overlap of K-M and COX analysis (Additional file 1: Table S1). To narrow down to the best suited CpGs, we used the following additional criteria: (1) Sites should reveal relatively high variation in DNAm across different AML samples (as a higher DNAm range facilitates more reliable discrimination); (2) DNAm levels should be similar in different types of blood cells (to avoid bias by blood counts) [10]; and (3) there should be no correlation with age. In fact, many CpGs reveal age-associated DNAm changes [11, 12] and age is a major confounding factor for OS. Therefore, we excluded CpGs with clear DNAm changes upon aging. Using these parameters, we identified four candidate CpGs (Additional file 1: Table S1; Fig. 1c, d; Additional file 1: Figures S1–S3), which were tested in an independent dataset of Qu et al. [13]. Only one CpG site, positioned in the 5’UTR of complement component 1 subcomponent R (*C1R,* cg08799922), revealed significant association with OS (K-M analysis: *P* = 0.009; COX analysis: *P* = 0.012; Fig. 1e, f). We then designed a pyrosequencing assay for site-specific analysis at this genomic location (Additional file 1: Figure S4). Pyrosequencing results of 84 AML patients were stratified by the threshold of 27 % DNAm at cg08799922 (*C1R*), corresponding to the median beta-value in the training set (TCGA). Again, there was a clear association with OS (K-M analysis: *P* = 0.012; COX analysis: *P* = 0.035; Fig. 1g, h).

### Epigenetic co-regulation of *C1R* with other genomic regions and correlation with gene expression

**Fig. 2:**
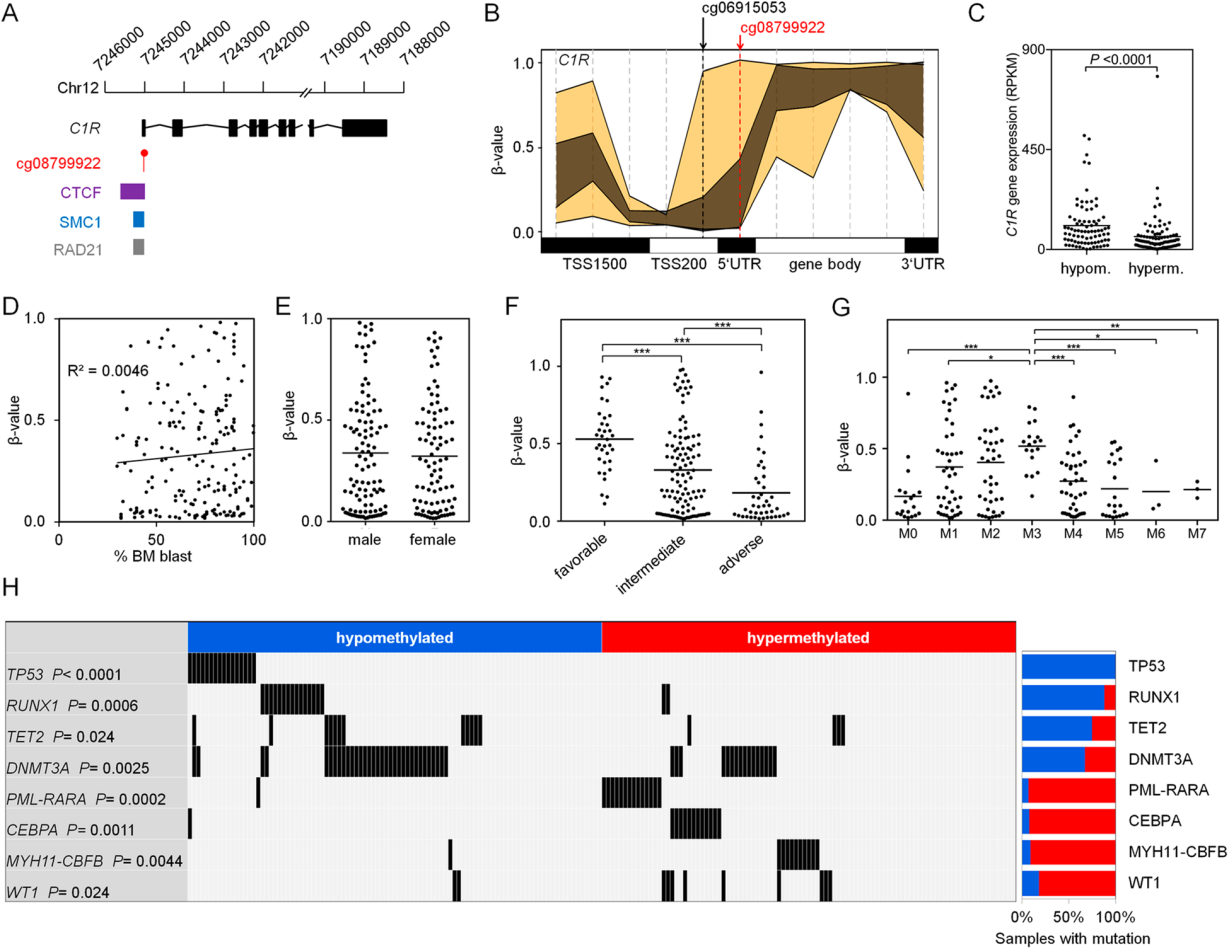
DNA-methylation in *C1R* is associated with clinical parameters. **a** The CpG site cg08799922 is positioned in a regulatory region in the *C1R* gene, with binding sites of the architectural protein CTCF and cohesin components SMC1 and RAD21 in close proximity [20]. **b** Beta-value range of 194 AML patients [9] (*yellow*) and 656 healthy individuals [19] (*brown*) at 11 CpGs that are associated with *C1R*. Particularly, our selected CpG cg08799922 (in 5’UTR; *red*) and another site cg06915053 (first upstream in TSS200) are associated with OS. **c** Overall, AML patients [9] with lower DNAm (<27 % DNAm level) at cg08799922 (*C1R*) have higher gene expression of *C1R*. Correlation of DNAm at cg08799922 (*C1R*) with bone marrow blasts (**d**), gender (**e**), cytogenetic risk (**f**), and AML FAB-subtype classification (**g**). *P < 0.01, **P < 0.001, and ***P < 0.0001 by Mann-Whitney test. **h** Enrichment of specific mutations according to DNAm level at *C1R* (*P* values: hypergeometric distribution)

The serine protease *C1R* catalyzes the initial event of the classical complement pathway, which is crucial for innate defense mechanisms against pathogens and altered-self cells [14]. The CpG site is located in the 5’UTR region of *C1R* and may interfere with a CCCTC-binding factor (CTCF) and cohesin-binding site (Fig. 2a) [15]. Differential DNAm in the neighboring genomic region is of prognostic relevance too, as another upstream site of cg08799922 was also among the initial 60 CpGs from the K-M estimate (Fig. 2b; cg06915053, adjusted *P* = 0.047). Furthermore, neighboring CpGs in the pyrosequencing assay revealed similar correlation with OS as well (Additional file 1: Figure S5). To determine whether or not DNAm at *C1R* (cg08799922) was co-regulated with other genomic regions, we performed linear correlation of DNAm with all CpGs from the 450K BeadChip: 1448 CpGs correlated (Pearson’s *R* >0.5), and these were particularly located in homeobox genes (Additional file 1: Figure S6). This co-regulation of *C1R* and *HOX* regions might be mediated by chromatin loops via CTCF and cohesin [16]. Furthermore, we analyzed if higher methylation of *C1R* entails down-regulation of the corresponding gene, but there was only a moderate association from RNA-seq data (*R* = 0.35, Fig. 2c). However, expression of 82 genes throughout the genome correlated with DNAm in *C1R* and were linked to developmental processes (*R* >0.5; Additional file 1: Figure S7). Taken together, DNAm of *C1R* does not seem to be relevant for functional changes in AML per se; it rather resembles a biomarker that reflects reorganization of chromatin in a subset of AML patients.

### Association of DNA-methylation at *C1R* with clinical parameters

DNAm levels in *C1R* were subsequently compared with clinical parameters of the TCGA dataset [9]: there was no clear association with blast counts (*R* = 0.0046; Fig. 2d) and no gender difference (Mann-Whitney *P* = 0.82; Fig. 2e). AML samples with favorable cytogenetic risk score (Fig. 2f) and AML subtype M3 (Fig. 2g) revealed significantly higher DNAm in *C1R*. Multivariate analysis of OS considering age, gender, bone marrow blast count, and FAB classification demonstrated that DNAm at *C1R* can support risk stratification (Additional file 1: Table S2). If alternatively only DNAm in *C1R*, cytogenetic risk score, and molecular risk score were included as parameters for multivariate analysis, the relevance of *C1R* was also significant (Additional file 1: Table S3). However, if all parameters were combined into one multivariate model, only age and molecular risk score were classified as relevant parameters (Additional file 1: Table S4). To further evaluate if the prognostic relevance of DNAm in *C1R* is independent from cytogenetic risk groups, we performed K-M analysis within individual cytogenetic groups. DNAm at *C1R* revealed significant association with OS in the intermediate group of TCGA (K-M analysis: *P* = 0.015), and the same trend was also observed in the other groups (in TCGA and pyrosequencing datasets; Additional file 1: Figure S8). These results indicate that DNAm in *C1R* might be of independent prognostic relevance, but this needs to be further validated in larger cohorts. Furthermore, higher methylation at *C1R* was associated with significantly less mutations in *TP53*, *RUNX1*, *TET2*, and *DNMT3A*, whereas mutations in *CEBPA* and *WT1* and the translocations *PML-PARA* and *MYH11-CBFB* were significantly enriched (Fig. 2h; Additional file 1: Figure S9). Thus, it can be speculated that either DNAm at *C1R* is influenced by these mutations or vice versa [12, 17]. Because of the strong interaction of DNAm at *C1R* with prognostic relevant mutations, we performed an additional Kaplan-Meier analysis for patients without the above mentioned mutations. In this subset of patients, DNAm of *C1R* was also indicative for OS (*P* = 0.036; Additional file 1: Figure S10).

### Analysis of DNA-methylation at *C1R* in various other types of cancer

To estimate if DNAm in *C1R* might also be a suitable biomarker for OS in other types of cancer, we utilized 5699 DNAm profiles of 25 different types of tumor from TCGA. In fact, significant association in COX analysis was also observed for kidney renal papillary carcinoma (*P* = 0.00009), low-grade glioma (*P* = 0.0002), skin cutaneous melanoma (*P* = 0.002), hepatocellular carcinoma *(P* = 0.026), and glioblastoma (*P* = 0.042; Additional file 1: Table S5). These results indicate similar association of DNAm in *C1R* with OS in other tumors, but the prognostic relevance was found to be particularly predominant in patients with AML.

## Conclusions

In this study, we demonstrate that DNAm at a single CpG site in *C1R* is indicative for overall survival in AML. In contrast to previously described epigenetic signatures [3, 7, 18], our method is based on only one CpG site. It is unclear if *C1R* plays a functional role for leukemia development or if it is differentially methylated in leukemia initiating cells, but this is not a prerequisite for biomarkers. Our data indicate that DNAm of *C1R* may reflect global chromatin reorganization in a subset of AML patients, and this may contribute to specific genomic mutations or vice versa [12, 17]. We have demonstrated that DNAm of *C1R* was also of prognostic relevance in cytogenetic normal AMLs, in subsets with defined cytogenetic risk score, and in subsets without specific genomic mutations. These results suggest that DNAm in *C1R* is of independent prognostic significance, but further validation with larger datasets is required to ultimately substantiate the clinical potential. Many cytogenetic aberrations are routinely analyzed in AML diagnostics, but the importance of epigenetic biomarkers should not be neglected, considering that AML develops by means of genetic and epigenetic changes as well. Complementation of epigenetic biomarkers to existing genetic biomarkers could provide a balanced and more accurate diagnostic approach. Furthermore, it is conceivable that DNAm in *C1R* is also indicative for treatment response to specific drugs, particularly for demethylating agents, and this needs to be addressed in future studies. Either way, analysis of DNAm at a unique CpG site provides a very simple and robust biomarker to complement risk assessment in AML.

## Study design

### DNAm profiles and bioinformatics analysis

We used publically available DNAm profiles (all based on HumanMethylation450K BeadChip) of AML patients from The Cancer Genome Atlas (TCGA) [9] and a study of Qu et al. (GSE58477) [13], of healthy individuals (GSE40279, GSE35069) [10, 19], and of 25 other types of cancer (TCGA). For Kaplan-Meier estimation of overall survival, we stratified samples by median DNAm levels and adjusted the results for multiple testing (log-rank test calculated in R). Alternatively, we calculated COX regression with OS for each individual CpG site in R.

### Blood samples and pyrosequencing

Blood samples were taken from 40 healthy donors and 84 AML patients after written consent according to the guidelines of the ethics committee of the Medical Faculty of the RWTH Aachen (Permit Number: EK206/09). Patient characteristics are summarized in Additional file 1: Table S6. Genomic DNA was isolated from blood, bisulfite converted, and analyzed by pyrosequencing (primers are provided in Additional file 1: Table S7). More detailed methods are described in the supplemental document.

### Availability of supporting data

The data sets supporting the results of this article are available at The Cancer Genome Atlas portal (https://tcga-data.nci.nih.gov/tcga/) [9] and at Gene Expression Omnibus (http://www.ncbi.nlm.nih.gov/geo/) under the accession numbers GSE58477 [13], GSE40279 [19], and GSE35069 [10].

## Additional file

Additional file 1:
**PDF with all supplemental data.** This file contains additional details on methods, Tables S1–S7, and Figures S1–S10. (PDF 1718 kb)

## Abbreviations

AML: Acute myeloid leukemia
C1R: Complement component 1 subcomponent R
CTCF: CCCTC-binding factor
DNAm: DNA-methylation
FAB: French-American-British classification of AML
K-M: Kaplan-Meier
OS: Overall survival
PSQ: pyrosequencing
TCGA: The Cancer Genome Atlas
