# Supplementary material for: DNA-methylation in C1R is a prognostic biomarker for acute myeloid leukemia
Source: Clin Epigenetics. 2015 Nov 4;7:116. doi: 10.1186/s13148-015-0153-6 (PMC4632269; doi:10.1186/s13148-015-0153-6)
Supplement: Additional file 1: — PDF with all supplemental data. This file contains additional details on methods, Tables S1–S7, and Figures S1–S10. (PDF 1718 kb) [file 13148_2015_153_MOESM1_ESM.pdf]

Supplemental Data

DNA-Methylation in *C1R* is a Prognostic Biomarker for Acute Myeloid Leukemia

Tanja Božić[1,2,*], Qiong Lin[1,2,*], Joana Frobel[1,2], Stefan Wilop[3], Melanie Hoffmann[3], Carsten Müller-Tidow[4], Tim H. Brümmendorf[3], Edgar Jost[3] and Wolfgang Wagner[1,2]

[1] Helmholtz-Institute for Biomedical Engineering, Stem Cell Biology and Cellular Engineering, University Hospital of the RWTH Aachen, Aachen, Germany; [2] Institute for Biomedical Engineering - Cell Biology, University Hospital of the RWTH Aachen, Aachen, Germany; [3] Department of Hematology, Oncology, Hemostaseology and Stem Cell Transplantation, University Hospital of the RWTH Aachen, Aachen, Germany; [4] Department of Hematology and Oncology, University of Halle, Halle, Germany

* These authors contributed equally to this work

Table of Content

Study Design 2

DNAm profiles used in this study 2

Bioinformatic analysis and statistics 2

Blood samples and pyrosequencing 2

Analysis of gene expression profiles..3

Supplemental Tables..3

Table S1. Significant CpGs in Kaplan-Meier analysis and COX model. 3

Table S2. Multivariate COX analysis of overall survival including clinical parameters. 4

Table S3. Multivariate COX analysis of overall survival including cytogenetic/molecular risk scores. 4

Table S4. Multivariate COX analysis of overall survival including clinical parameters and risk scores. 4

Table S5. DNA-methylation in *C1R* indicates overall survival in other tumors. 5

Table S6. Patient characteristics for pyrosequencing validation. 6

Table S7. Primers used in the pyrosequencing assay. 6

Supplemental Figures...7

Figure S1. COX model analysis of overall survival in AML. 7

Figure S2. Three alternative CpG sites of initial screen. 7

Figure S3. Impact of blood counts and age on DNA-methylation levels. 8

Figure S4. Design of pyrosequencing assay for *C1R*. 9

Figure S5. Kaplan-Meier plots of neighboring CpGs in the pyrosequencing assay. 9

Figure S6. Association of DNA-methylation at *C1R* with methylation. 10

Figure S7. Gene ontology of 82 genes that correlate with DNA-methylation at *C1R*. 10

Figure S8. Kaplan-Meier plots of AML samples with different cytogenetic risk scores. 11

Figure S9. Other frequent mutations are not associated with DNA-methylation at *C1R*. 12

Figure S10. Kaplan-Meier plot of AML samples without relevant mutations. 12

References of supplemental data 12

Study Design

DNAm profiles used in this study

For this study, we used DNAm profiles generated with Illumina HumanMethylation450K BeadChip platform, which covers about 480,000 CpG sites at single base resolution (including 99% of RefSeq genes and 96% of CpG islands) [1]. CpG sites located in sex chromosomes, single nucleotide polymorphisms (SNPs), and those with missing values in several samples were excluded resulting in about 390,000 CpG sites. Beta-values ranging from 0 (non-methylated) to 1 (100% methylation) are provided for each CpG site. Affiliation of CpG sites with gene regions or CpG islands was used as described in detail before [2]. We used DNAm profiles of 194 AML patients from TCGA (https://tcga-data.nci.nih.gov/tcga/) [3] for training; 62 cytogenetic normal AML patients from the Karolinska study (GSE58477) [4] for validation; 656 healthy blood samples as controls (GSE40279) [5], and 60 profiles of different types of blood cells (GSE35069) [6] for blood composition association. Furthermore, we analyzed DNAm of *C1R* in 5699 DNAm profiles of 25 other types of cancer (all TCGA; supplemental Table 5).

Bioinformatic analysis and statistics

Two approaches were used for initial selection to correlate methylation of a single CpG site with survival data – Kaplan-Meier (K-M) and COX proportional hazards model (both adjusted for multiple testing, P values <0.05). For K-M analysis the samples were stratified into two groups according to the median DNAm level of each individual CpG site (hypomethylated and hypermethylated), whereas COX model provides a direct correlation with overall survival (OS). We defined OS as the survival time from the first day of diagnosis to day of death by any cause. Deceased patients are considered to have an event, whereas patients with incomplete survival information are marked as censored (hyphens in survival plots). Kaplan-Meier plots and Mann-Whitney statistics were generated with GraphPad Prism 6.05 (GraphPad Software, San Diego, USA). Association of CpG location with its genomic environment was analyzed with the UCSC Genome Browser, Human Genome Assembly GRCh37/hg19 (http://genome.ucsc.edu/). Comparison of DNAm with clinical parameters was performed two-sided using the SAS software package version 9.1.3 (SAS Institute Inc., Cary, NC, USA). Multivariate analysis was performed in R.

Blood samples and pyrosequencing

Genomic DNA was isolated from peripheral blood of 40 healthy donors and of bone marrow aspirates of 84 AML patients using the QIAamp DNA Blood Mini Kit (Qiagen, Hilden, Germany). One µg of DNA was sodium bisulfite-converted with the EZ DNA Methylation Kit (Zymo Research, Irvine, USA). The region of interest was amplified by PCR and sequenced on a PyroMark Q96 ID System with a gene specific sequencing primer. PCR and sequencing primers were designed with Pyrosequencing Assay Design Sofware 1.0 (Biotag AB, Uppsala, Sweden; supplemental Table 7).

Analysis of gene expression profiles

RNA-sequencing data for AML samples was downloaded from TCGA (https://tcga-data.nci.nih.gov/tcga/). Gene expression profiles with corresponding DNAm profiles were considered for further analysis (UNC IlluminaHiSeq_RNASeqV2; n = 170). Furthermore, we correlated DNAm at cg08799922 (*C1R*) with differential gene expression of 20,000 genes (Pearson's correlation). Gene ontology (GO) analysis was performed with the online tool David GoTerm_BP_All (https://david.ncifcrf.gov/).

Supplemental Tables

Table S1. Significant CpGs in Kaplan-Meier analysis and COX model.

CpG ID	Kaplan-Meier (adjusted *P value*)	Cox (adjusted *P value*)	Chromosome	Gene Name	Poor Prognosis
cg26325912	0.0128	0.0151	5	-	Hypermethylation
cg08799922	0.0181	0.0150	12	*C1R*	Hypomethylation
cg10977795	0.0201	0.0291	6	*SYNJ2*	Hypomethylation
cg17576375	0.0217	0.0097	2	*TGFBRAP1*	Hypermethylation
cg1632789	0.0256	0.0120	13	*EPSTI1*	Hypomethylation
cg21210041	0.0292	0.0343	17	*MYO18A*	Hypermethylation
cg26714230	0.0337	0.0103	13	*EPSTI1*	Hypomethylation
cg22725197	0.0354	0.0150	1	*UBE2J2*	Hypomethylation
cg25243766	0.0354	0.0080	11	-	Hypomethylation
cg09288989	0.0383	0.0380	12	*SETD1B*	Hypomethylation
cg02194129	0.0429	0.0118	14	*XRCC3*	Hypomethylation
cg12516875	0.0429	0.0179	22	-	Hypomethylation
cg00861646	0.0433	0.0118	4	-	Hypomethylation
cg19893929	0.0452	0.0118	2	-	Hypermethylation
cg11004284	0.0452	0.0063	3	*METTL6;EAF1*	Hypomethylation
cg01446217	0.0452	0.0132	9	*ERMP1*	Hypermethylation
cg19069882	0.0452	0.0063	11	*MPZL3*	Hypermethylation
cg13074055	0.0452	0.0292	14	-	Hypomethylation
cg14172849	0.0452	0.0246	14	*XRCC3*	Hypomethylation
cg05776053	0.0460	0.0027	2	-	Hypermethylation
cg27170268	0.0460	0.0435	14	*XRCC3*	Hypermethylation
cg22695532	0.0472	0.0355	1	-	Hypermethylation
cg16306870	0.0472	0.0088	3	*C3orf21*	Hypomethylation
cg13973002	0.0472	0.0120	19	*GLTSCR1*	Hypermethylation
cg12359279	0.0472	0.0119	21	*MX1*	Hypermethylation
cg01021169	0.0472	0.0155	22	*ASCC2*	Hypermethylation

Four CpGs were identified as potential candidates for further analysis as indicated in the text (highlighted in red). Poor prognosis is either associated with hypo- or hypermethylation (stratified by median DNAm level) at the corresponding CpG site.

Table S2. Multivariate COX analysis of overall survival including clinical parameters.

Parameter	Coefficient	Hazard ratio	95% Confidence interval	*P* value
cg08799922 (***C1R***)	-0.806	0.447	[0.20-0.98]	0.045
age	0.033	1.033	[1.02-1.05]	$1.4*10^{-5}$
gender	-0.174	0.840	[0.59-1.19]	0.332
BM Blast*	0.005	1.005	[0.99-1.01]	0.316
FAB*	0.023	1.023	[0.92-1.13]	0.659

Multivariate COX *P*= $1.7*10^{-7}$; *BM Blast = bone marrow blast count; FAB = French-American-British classification.

Table S3. Multivariate COX analysis of overall survival including cytogenetic/molecular risk scores.

Parameter	Coefficient	Hazard ratio	95% Confidence interval	*P* value
cg08799922 (***C1R***)	-1.093	0.335	[0.16-0.69]	0.003
Cytogenetic risk	-0.104	0.901	[0.55-1.48]	0.679
Molecular risk	0.550	1.734	[1.06-2.85]	0.030

Multivariate COX P= $5.1*10^{-7}$.

Table S4. Multivariate COX analysis of overall survival including clinical parameters and risk scores.

Parameter	Coefficient	Hazard ratio	95% Confidence interval	*P* value
cg08799922 (***C1R***)	-0.303	0.738	[0.33-1.66]	0.463
age	0.033	1.034	[1.02-1.05]	9.19E-06
gender	-0.248	0.780	[0.55-1.11]	0.171
BM Blast*	0.004	1.004	[0.99-1.01]	0.350
FAB*	0.034	1.035	[0.93-1.15]	0.517
Cytogenetic risk	-0.117	0.890	[0.51-1.56]	0.684
Molecular risk	0.618	1.855	[1.07-3.21]	0.027

Multivariate COX *P*= $4.7*10^{-9}$; *BM Blast = bone marrow blast count; FAB = French-American-British classification. Combination of all of these clinical, cytogenetic and molecular parameters in one multivariate analysis reduced the relevance of DNAm at *C1R*.

Table S5. DNA-methylation in *C1R* indicates overall survival in other tumors.

Tumor type	Shortcut	No. of DNAm profiles	Cox *P* value	K-M *P* value	Median DNAm level
Acute myeloide leukemia	LAML	194	$4.9*10^{-6}$	$9.4*10^{-8}$	0.278
Kidney renal papillary cell CA	KIRP	172	$8.9*10^{-5}$	0.1499	0.031
Lower grade glioma	LGG	412	0.0002	0.0009	0.225
Skin cutaneous melanoma	SKCM	358	0.0027	0.0013	0.065
Hepatocellular carcinoma	LIHC	164	0.0264	0.1178	0.032
Glioblastoma multiforme	GBM	127	0.0421	0.8563	0.037
Pancreatic adenocarcinoma	PAAD	79	0.0504	0.2785	0.072
Colon adenocarcinoma	COAD	289	0.0728	0.5417	0.052
Head and Neck squamous cell CA	HNSC	421	0.1594	0.5115	0.077
Esophageal carcinoma	ESCA	134	0.1882	0.7099	0.045
Thyroid carcinoma	THCA	500	0.2405	0.7508	0.024
Stomach adenocarcinoma	STAD	295	0.2535	0.5450	0.087
Lung adenocarcinoma	LUAD	393	0.2556	0.5070	0.102
Adrenocortical carcinoma	ACC	78	0.3634	0.0211	0.022
Prostate adenocarcinoma	PRAD	281	0.3714	0.1006	0.022
Sarcoma	SARC	115	0.3968	0.9347	0.022
Bladder Urothelial Carcinoma	BLCA	218	0.4019	0.4359	0.033
Endocervical adenocarcinoma	CESC	195	0.4106	0.3528	0.055
Renal clear cell carcinoma	KIRC	294	0.5789	0.7917	0.038
Uterine carcinosarcoma	UCS	57	0.5861	0.6305	0.027
Pheochromocyt. & paraganglioma	PCPG	97	0.5968	0.2882	0.027
Lung squamous cell carcinoma	LUSC	280	0.6130	0.4856	0.067
Rectum adenocarcinoma	READ	96	0.6448	0.7524	0.035
Uterine corpus endometrial CA	UCEC	384	0.7223	0.7044	0.025
Kidney chromophobe	KICH	66	0.8177	0.4502	0.021

Kaplan-Meier (K-M) analysis was stratified by the median DNAm level in the corresponding dataset; CA = carcinoma; *P* values are not adjusted for multiple testing as they are specifically tested for *C1R*. Significant results are highlighted in red.

Table S6. Patient characteristics for pyrosequencing validation.

Number of patients	Total 84
**Gender**	
Male	42 (50%)
Female	42 (50%)
**Age**	
<60 years	41 (49%)
>60 years	43 (51%)
Age mean (range)	59 (28-89)
**FAB***	
M0	5 (5.9%)
M1	30 (35.7%)
M2	13 (15.5%)
M3	3 (3.6%)
M4	24 (28.6%)
M4Eos*	2 (2.4%)
M5	4 (4.8%)
NA*	3 (3.6%)
**Cytogenetic risk**	
Favorable	12 (14.3%)
Intermediate	49 (58.3%)
Adverse	16 (19%)
NA*	7 (8.3%)
**Preexisting myelodysplastic syndrome**	25 (29.8%)

*FAB = French-American-British classification; M4Eo = M4 with eosinophilia; NA = not available.

Table S7. Primers used in the pyrosequencing assay.

Primer	Sequence
Forward	5´-TGTTGTGTATGACGCGGGTTTTTTTTGTATATAGT-3´
Reverse	5´-Biotin-AATTACCCATCACCCTTACTCACATTTC-3´
Sequencing	5´-CGCGGGTTTTTTTTGTATATAG-3´

Supplemental Figures

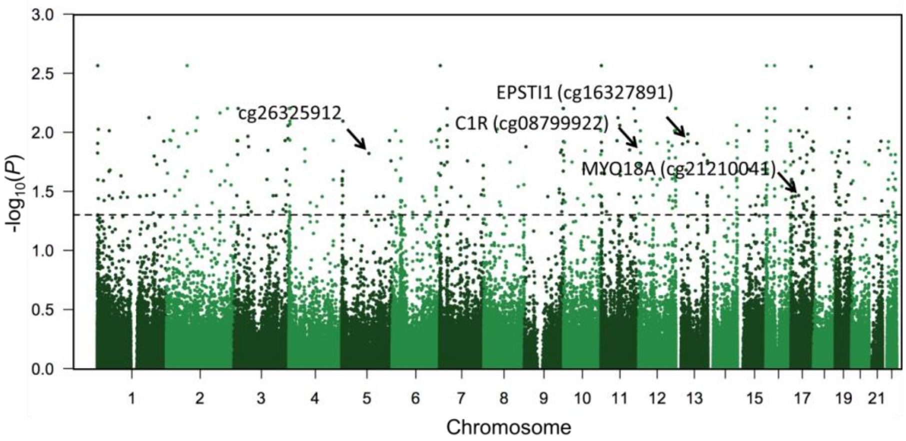

Figure S1. COX model analysis of overall survival in AML.

Manhattan plot depicts adjusted *P* values for each of the 390,000 CpG sites in 194 AML patients [3]. 418 CpG sites revealed adjusted *P*< 0.05 (dashed line) in COX analysis of overall survival and they were distributed across all chromosomes. Arrows highlight four shortlisted CpGs.

Figure S2. Three alternative CpG sites of initial screen.

(A) Beta-value distribution of 656 healthy controls [5] and 194 AML samples [3] for the alternative CpG sites: cg26325912 located in the non-coding region of chromosome 5, cg16327891 in *EPSTI1* (epithelial stromal interaction 1), and cg21210041 in *MYO18A* (myosin XVIIIA). Hypo- and hypermethylation as compared to the median DNAm level are depicted in blue and red, respectively. (B) Survival plots for these CpG sites in 194 AML samples of TCGA (stratified by corresponding median DNAm level).

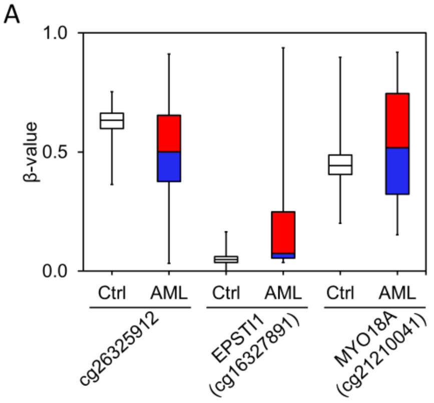

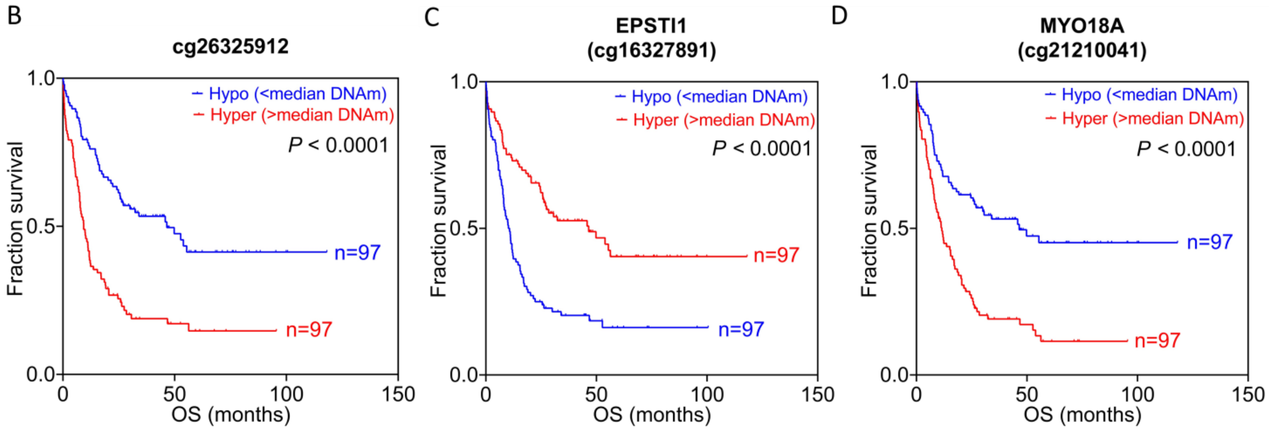

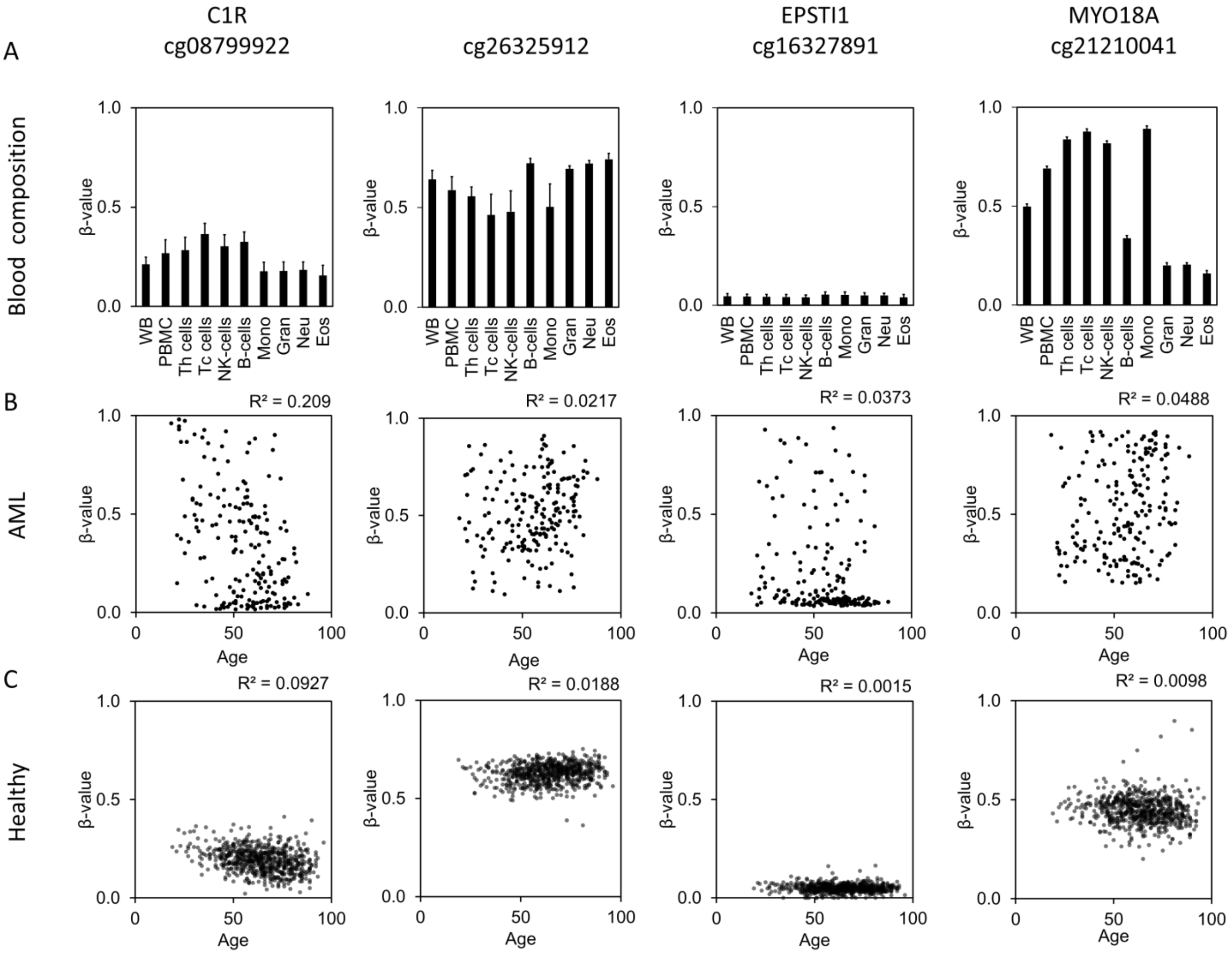

Figure S3. Impact of blood counts and age on DNA-methylation levels.

We analyzed the association of DNAm levels at the 4 selected CpG sites with potentially confounding parameters. (A) DNAm levels were analyzed in HumanMethylation450K BeadChip data of specific blood cell types from 6 healthy individuals (mean ± s.d.) [6]. The DNAm level in *C1R* was only slightly higher in lymphocytes than in myeloid cells indicating that blood counts do not have major impact on this CpG site. (WB= whole blood, PBMC= peripheral blood mononuclear cells, Th= T-helper cells, Tc= T-cytotoxic cells, Mono= monocytes, Gran= granulocytes, Neu= neutrophils, Eos= eosinophils). (B) There was a moderate association with patient age in 194 AML samples from TCGA [3]. However, in malignant tissue – particularly in AML – we have demonstrated that age-associated DNAm is coherently modified and does not reflect chronological age of the patient [7]. (C) In fact, none of the four selected CpGs revealed clear age-associated DNAm changes in 656 DNAm profiles of healthy donors [5].

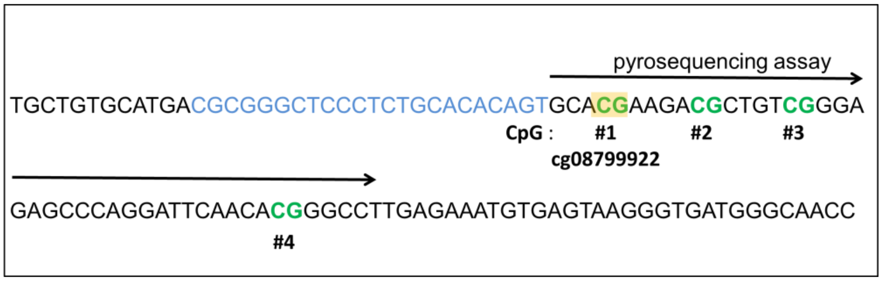

Figure S4. Design of pyrosequencing assay for *C1R*.

The genomic sequence that is amplified in bisulfite converted DNA by the PCR primers is demonstrated. Location of the sequencing primer is highlighted in blue and the direction of the pyrosequencing reaction is indicated with a black arrow. This assay covers four CpG sites (green), with the relevant CpG site cg08799922 (*C1R*) first in the sequence (highlighted in yellow).

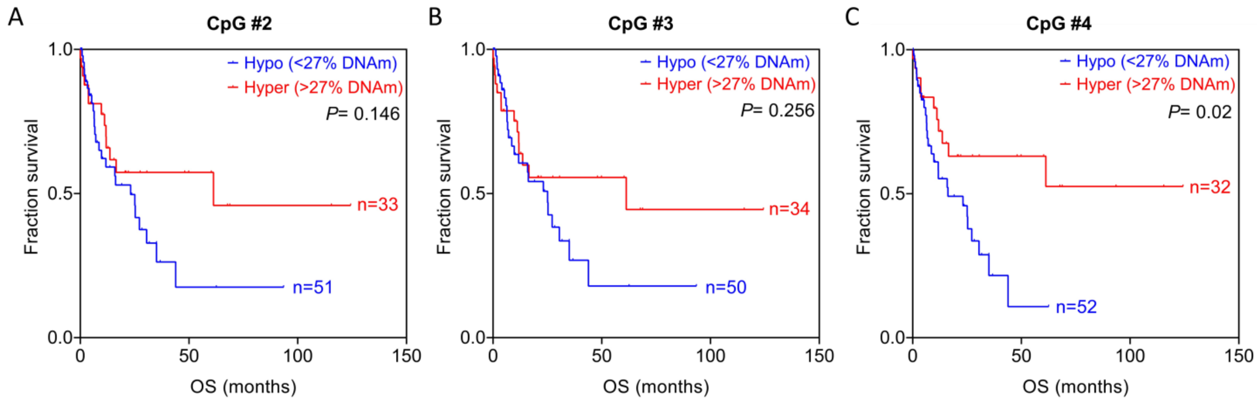

Figure S5. Kaplan-Meier plots of neighboring CpGs in the pyrosequencing assay.

Stratification of 84 AML samples by DNAm level of 27% for the downstream CpG #2 (A), CpG #3 (B) and CpG #4 (C; see supplemental Figure 4 for further details). These results demonstrate that DNAm at the neighboring CpGs of cg08799922 seem to be co-regulated – and thus is associated with overall survival too.

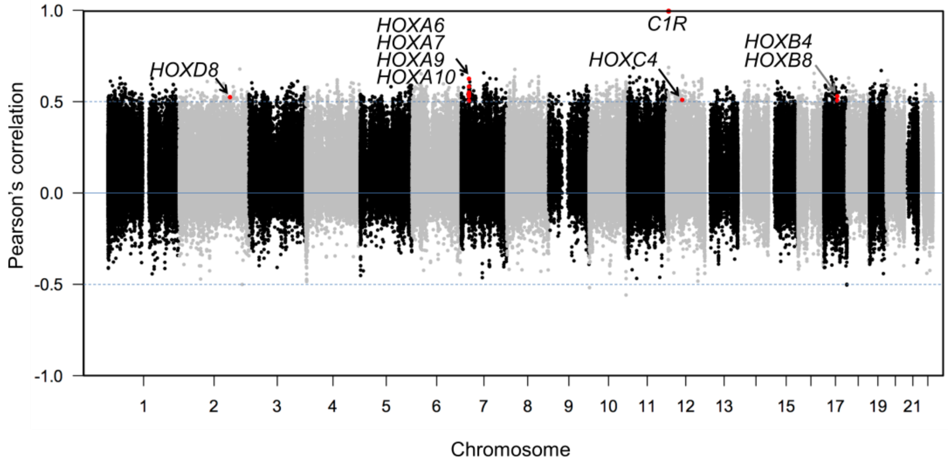

Figure S6. Association of DNA-methylation at *C1R* with methylation.

Pearson's correlation of DNAm at cg08799922 (*C1R*) with 390,000 CpG sites from TCGA [3]. In total, 1,448 CpGs revealed positive linear correlation (*R* >0.5) with cg08799922 (*C1R*). Relevant CpGs were located in genes of the HOX-cluster (often multiple CpGs per HOX gene). In contrast, only 5 CpGs revealed negative correlation with DNAm at *C1R* (*R* <-0.5).

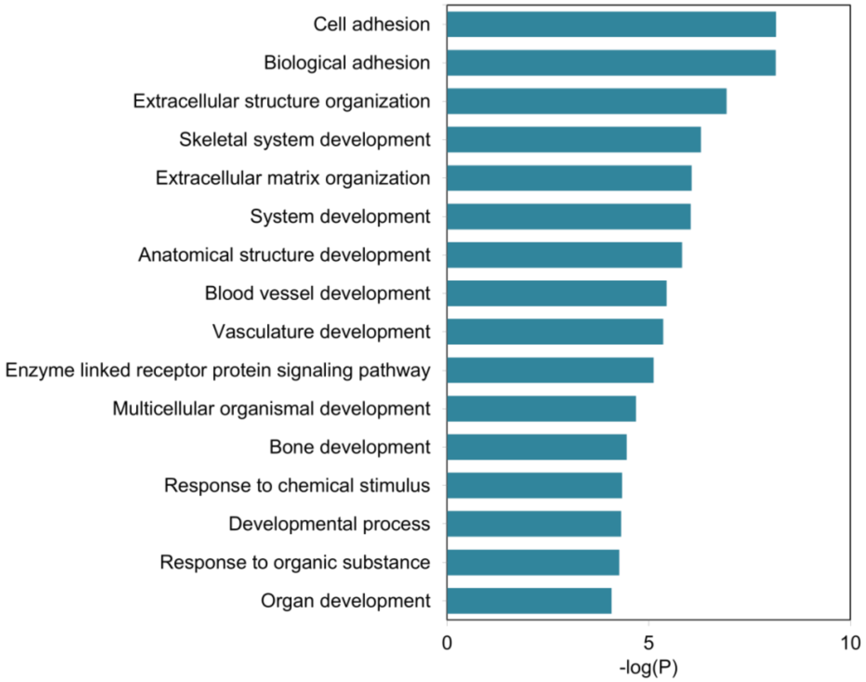

Figure S7. Gene ontology of 82 genes that correlate with DNA-methylation at *C1R*.

Genes that correlated with DNAm at cg08799922 (*C1R; R* >0.5) are enriched in developmental categories.

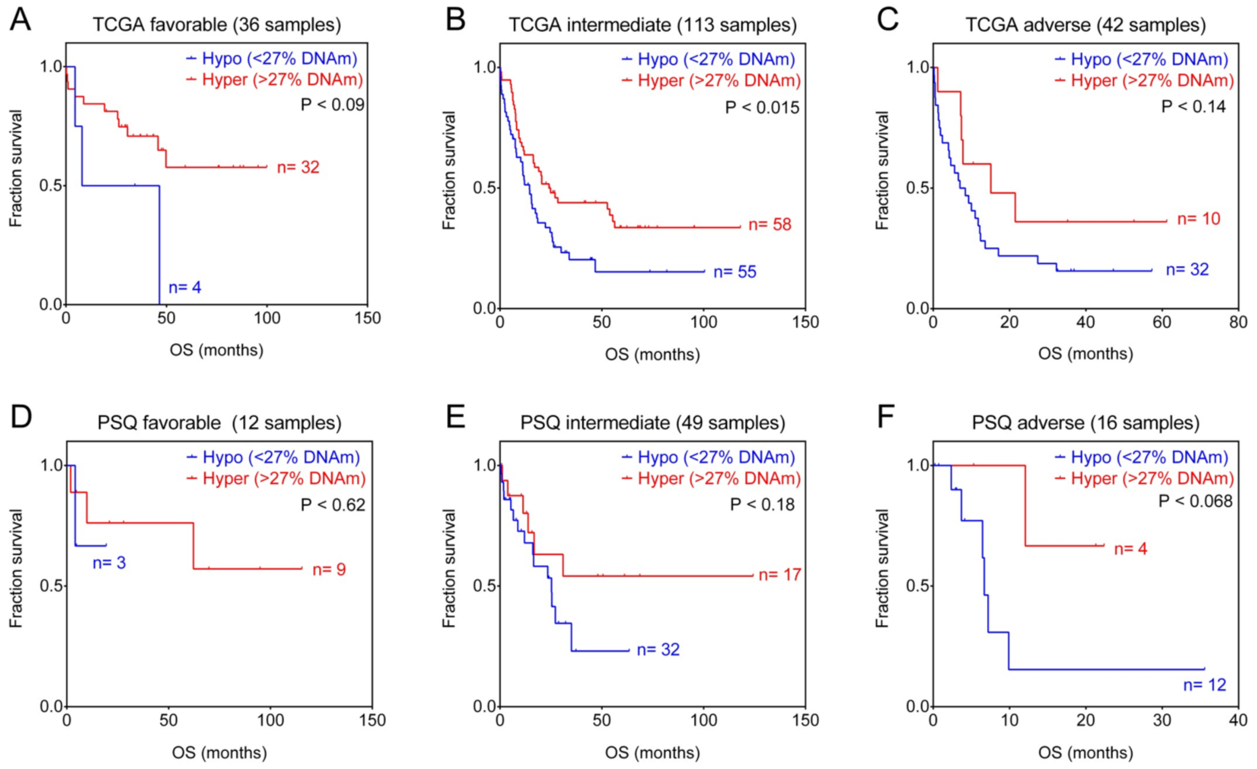

Figure S8. Kaplan-Meier plots of AML samples with different cytogenetic risk scores.

Association of DNAm in *C1R* with overall survival was estimated in AML subsets with either favorable, intermediate, or adverse cytogenetic risk score. This analysis was performed for 191 AML samples from TCGA (A-C) [3] and for 77 AML samples that were analyzed by pyrosequencing (PSQ; D-F; all with known cytogenetic risk scores). Samples were stratified by 27% DNAm at cg08799922 (*C1R*). DNAm at *C1R* revealed significant association with OS for the intermediate risk group in TCGA data (B). The same trend was also observed in all other groups, but the results did not reach statistical significance – probably due to the relatively small numbers of samples in these subsets.

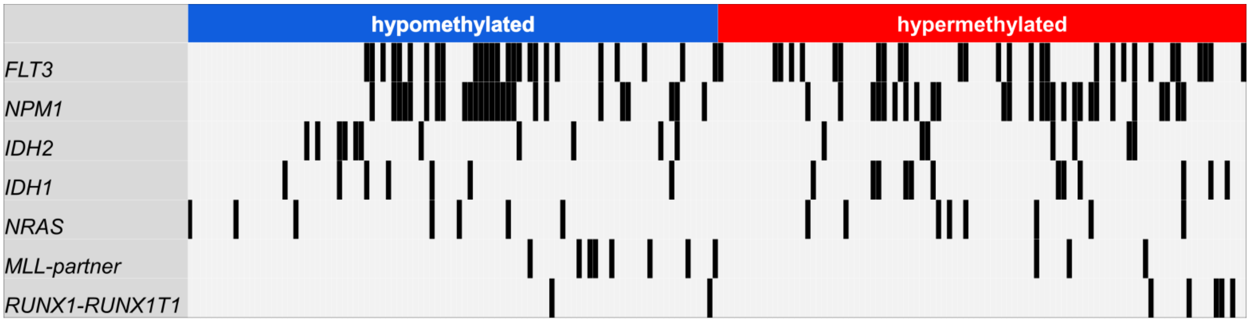

Figure S9. Other frequent mutations are not associated with DNA-methylation at *C1R*.

In analogy to the analysis of Figure 1H we have analyzed association of DNA-methylation at *C1R* with all other mutations that occurred in at least 10 samples in TCGA. We did not observe significant enrichment in the *C1R* hypo- and hypermethylated subgroups for mutations in *FLT3*, *NPM1*, *IDH1/2* and *NRAS,* or chromosome aberrations *MLL-partner* and *RUNX1-RUNX1T1* (194 AML samples were stratified by median DNAm level at cg08799922).

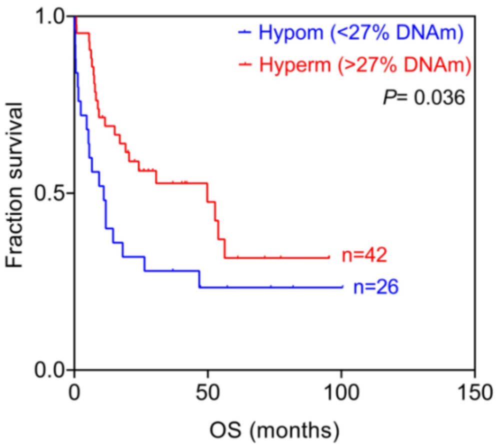

Figure S10. Kaplan-Meier plot of AML samples without relevant mutations.

Kaplan-Meier plot of 68 AML samples (TCGA) [3] without any of the mutations mentioned in Figure 2H revealed a significant trend for OS. Samples were stratified by 27% of DNAm level at cg08799922 (*C1R*).

References of supplemental data

1. Bibikova M, Barnes B, Tsan C, Ho V, Klotzle B, Le JM, Delano D, Zhang L, Schroth GP, Gunderson KL, Fan JB, Shen R: High density DNA methylation array with single CpG site resolution. Genomics 2011; **98:** 288-295.
2. Sandoval J, Heyn HA, Moran S, Serra-Musach J, Pujana MA, Bibikova M, Esteller M: Validation of a DNA methylation microarray for 450,000 CpG sites in the human genome. Epigenetics 2011; **6:** 692-702.
3. Cancer Genome Atlas Research Network.: Genomic and epigenomic landscapes of adult de novo acute myeloid leukemia. N Engl J Med 2013; **368:** 2059-2074.
4. Qu Y, Lennartsson A, Gaidzik VI, Deneberg S, Karimi M, Bengtzen S, Hoglund M, Bullinger L, Dohner K, Lehmann S: Differential methylation in CN-AML preferentially targets non-CGI regions and is dictated by DNMT3A mutational status and associated with predominant hypomethylation of HOX genes. Epigenetics 2014; **9:** 1108-1119.
5. Hannum G, Guinney J, Zhao L, Zhang L, Hughes G, Sadda S, Klotzle B, Bibikova M, Fan JB, Gao Y, Deconde R, Chen M, Rajapakse I, Friend S, Ideker T, Zhang K: Genome-wide Methylation Profiles Reveal Quantitative Views of Human Aging Rates. Mol Cell 2013; **49:** 459-367.
6. Reinius LE, Acevedo N, Joerink M, Pershagen G, Dahlen SE, Greco D, Soderhall C, Scheynius A, Kere J: Differential DNA methylation in purified human blood cells: implications for cell lineage and studies on disease susceptibility. PLoS ONE 2012; **7:** e41361.
7. Lin Q, Wagner W: Epigenetic Aging Signatures Are Coherently Modified in Cancer. PLoS Genet 2015; **11:** e1005334.
